# Concentration of Phosphatidylserine Influence Rates of Insulin Aggregation and Toxicity of Amyloid Aggregates In Vitro

**DOI:** 10.1021/acschemneuro.3c00277

**Published:** 2023-06-06

**Authors:** Mikhail Matveyenka, Kiryl Zhaliazka, Dmitry Kurouski

**Affiliations:** † Department of Biochemistry and Biophysics, Texas A&M University, College Station, Texas 77843, United States; ‡ Department of Biomedical Engineering, Texas A&M University, College Station, Texas 77843, United States

**Keywords:** insulin, phosphatidylserine, oligomers, fibrils, AFM-IR

## Abstract

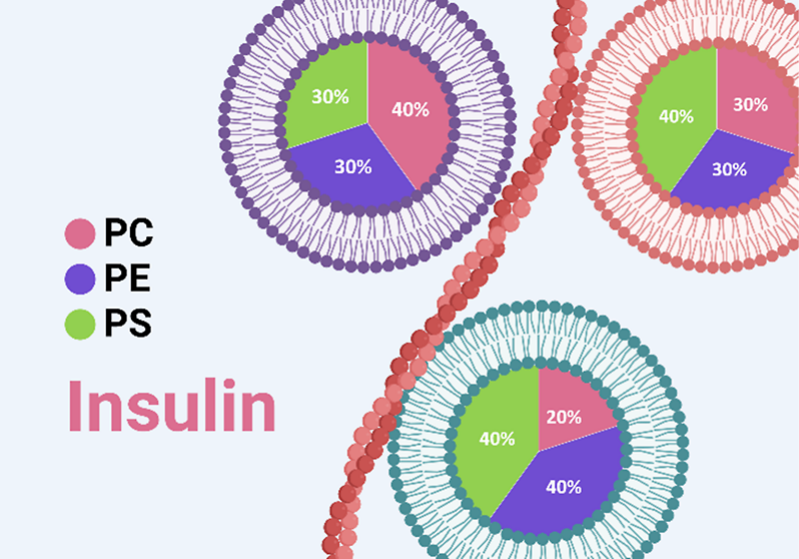

Phosphatidylserine (PS) is a negatively charged lipid that plays a critically important role in cell apoptosis. Under physiological conditions, PS is localized on the cytosolic side of plasma membranes via ATP-dependent flippase-mediated transport. A decrease in the ATP levels in the cell, which is taken place upon pathological processes, results in the increase in PS concentration on the exterior part of the cell membranes. PS on the outer membrane surfaces attracts and activates phagocytes, which trigger cell apoptosis. This programed irreversible cell death is observed upon the progressive neurodegeneration, a hallmark of numerous amyloid associated pathologies, such as diabetes type 2 and Alzheimer’s disease. In this study, we investigate the extent to which the rates of protein aggregation, which occurs upon amyloid pathologies, can be altered by the concentration of PS in large unilamellar vesicles (LUVs). We found that with an increase in the concentration of PS from 20 to 40% relative to the concentration of phosphatidylcholine and phosphatidylethanolamine, the rate of insulin aggregation, protein linked to diabetes type 2, and injection amyloidosis drastically increased. Furthermore, the concentration of PS in LUVs determined the secondary structure of protein aggregates formed in their presence. We also found that these structurally different aggregates exerted distinctly different cell toxicities. These findings suggest that a substantial decrease in cell viability, which is likely to take place upon aging, results in the increase in the concentration of PS in the outer plasma membranes, where it triggers the irreversible self-assembly of amyloidogenic proteins, which, in turn, causes the progressive neurodegeneration.

## Introduction

Abrupt aggregation of misfolded proteins is a hallmark of amyloid diseases, a large group of severe pathologies that include diabetes type 2, Alzheimer’s disease (AD), and Parkinson’s disease (PD).^1,2^ The abrupt aggregation yields protein oligomers, toxic species with a large variety of forms and sizes.^3−5^ Some of these oligomers can propagate into fibrils, long unbranched protein aggregates with a β-sheet secondary structure.^6−9^ Structural characterization of amyloid oligomers and fibrils is a challenging task, primarily because the high structural heterogeneity and transient nature of protein oligomers limit the use of cryo-electron microscopy and solid-state nuclear magnetic resonance, classical tools of structural biology, for elucidation of their secondary structure.^3−5,10,11^ Optical nanoscopy techniques, which include atomic force microscopy infrared (AFM-IR) and tip-enhanced Raman spectroscopy (TERS), can be used to overcome these limitations.^12−17^ In both AFM-IR and TERS, a metallized scanning probe can be positioned directly at the sample of interest.^18^ Next, in the case of AFM-IR, the probe is illuminated by pulsed tunable IR light. IR pulses include thermal expansions in the sample, which are reordered by the scanning probe and converted to the corresponding IR spectra.^19−21^ High sensitivity and nanometer spatial resolution of AFM-IR were utilized to reveal the secondary structure of amyloid fibrils,^12,14,15,22−24^ plant epicuticular waxes,^25,26^ liposomes,^27^ malaria-infected blood cells,^28^ polymers,^29^ and bacteria.^30−32^ In TERS, laser light is used to generate localized surface plasmon resonances at the apex of metallized scanning probe, which, in turn, enhance Raman scattering from molecules located directly under the probe.^21,33,34^ Using TERS, Kurouski and co-workers examined the secondary structure of insulin fibril polymorphs,^35,36^ whereas Bonhommeau and co-workers determined the nanoscale structural organization of amyloid β aggregates using this unique optical nanoscopy technique.^37^

A growing body of evidence suggests that lipids can play an important role in the aggregation of amyloidogenic proteins.^38−43^ For instance, Zhaliazka and co-workers demonstrated that zwitterionic phospholipids strongly inhibited lysozyme aggregation, whereas negatively charged lipids drastically accelerated lysozyme aggregation.^44^ Similar findings were recently reported by Matveyenka and co-workers.^38−43^ Specifically, it was found that phosphatidylcholine (PC) strongly inhibited insulin aggregation.^41−43^ However, negatively charged phosphatidic acid (PA) and cardiolipin (CL) drastically enhanced the rate of lysozyme aggregation.^40−43^ Galvagnion and co-workers found that lipids could facilitate α-Syn aggregation.^45−47^ Expanding upon this, Dou and co-workers discovered that both PC and PS not only altered the rates of α-Syn aggregation but also uniquely modified the secondary structure of protein oligomers formed in the presence of these lipids.^48,49^ Zhang and co-workers found that low levels of anionic lipids promoted the aggregation of amyloid precursor peptide (IAPP).^50^ IAPP aggregates formed in the presence of such lipids exhibited high membrane permeabilization potential. Zhang and co-workers also found that zwitterionic lipids did not alter the rate of IAPP aggregation, whereas cholesterol at or below physiological levels significantly decelerated protein aggregation as well as lowered the propensity of IAPP aggregates to cause membrane leakage. Avdulov and co-workers found that lipids could uniquely alter the structure of amyloid β1-40 (Aβ1-40) aggregates.^51^

Our group recently demonstrated that phosphatidylserine (PS) could alter the aggregation rate of insulin and lysozyme.^39^ Matveyenka and co-workers found that the rate of protein aggregation was determined by the degree of saturation of fatty acids in PS.^39^ Specifically, the equimolar presence of fully saturated PS enabled the strongest enhancement of the rate of insulin aggregation compared to PS with unsaturated fatty acids. It was also found that insulin and lysozyme aggregates formed in the presence of PS exerted significantly different cell toxicities compared to the fibrils grown in the lipid-free environment.^39,43,44^ Although the above-discussed findings can be valuable for the understanding of the role of PS in amyloidosis, they were obtained upon exposition of both insulin and lysozyme to the large unilamellar vesicles (LUVs) composed of PS alone.^39,43,44^ The question to ask is whether an increase in the concentration of PS relative to zwitterionic lipids, such as PC and phosphatidylethanolamine (PE), primary constituents of the plasma membranes of most eucaryotic cells, would alter the rate of protein aggregation, as was evident for PS itself. To answer this question, we used a set of biophysical and molecular biology techniques that allowed for the determination of the rate of protein aggregation, the elucidation of morphology, secondary structure, and the toxicity of protein aggregates grown in the presence of lipid mixtures and in the lipid-free environment.

## Results and Discussion

### Elucidation of the Relationship between Concentration of PS and the Rate of Insulin Aggregation

**Figure 1:**
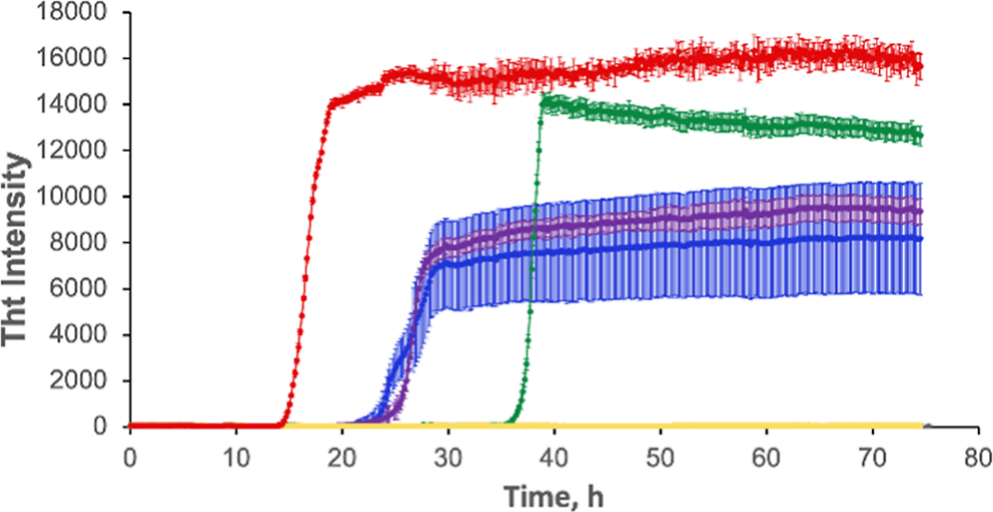
Increase in the concentration of PS in the lipid mixtures increases the aggregation rate of insulin. Averages of triplicates of ThT aggregation kinetics of insulin (Ins) in the lipid-free environment (red), insulin in the presence of LUVs of PC/PE/PS (40:40:20) (yellow), PC/PE/PS (30:40:30) (green), PC/PE/PS (20:40:40) (blue), and PC/PE/PS (30:30:40) (purple). All measurements were made in triplicate.

In the lipid-free environment, insulin exhibits a well-defined lag phase (*t*_lag_ = 13.3 ± 0.7 h) on which protein aggregates into small oligomers, Figure 1 and Table S1. It should be noted that *t*_lag_ corresponds to a 10% increase in the ThT intensity. Once their critical concentration is reached, protein oligomers rapidly propagate into fibrils, which result in the increase in ThT fluorescence.^39,43,44^ We found that in the presence of LUVs made of two zwitterions, PC and PE, as well as negatively charged PS at 40:40:20 molar ratios, insulin fibrillization was not observed for 75 h. These results are in good agreement with the previously reported results by Matveyenka and co-workers that showed that PC strongly decelerated insulin aggregation.^39,43^

We also found that an increase in the molar ratio of PS relative to PC/PE accelerated insulin aggregation. Specifically, we found that in the presence of an equimolar concentration of LUV with PC/PE/PS at 30:40:30 molar ratios, *t*_lag_ of insulin aggregation was found to be 36.7 ± 0.7 h, Figure 1 and Table S1. At the same time, if the molar ratio of PS was increased to PC/PE/PS (20:40:40) and PC/PE/PS (30:30:40), *t*_lag_ of insulin aggregation was found to be 24.4 ± 0.3 and 25.1 ± 0.6 h, respectively. These results demonstrate that an increase in the concentration of PS in the LUVs composed of PC and PE results in the increase in the rate of insulin aggregation. Our findings also suggest that a change in the relative concentrations of two zwitterions does not substantially alter the rate of insulin aggregation, which is consistent with the reported results for lysozyme by Zhaliazka and co-workers, according to which both PC and PE equivalently strongly inhibit protein aggregation.^44^

### Morphological and Structural Characterization of Insulin Aggregates Grown in the Presence of LUVs with Different Concentrations of PS

**Figure 2:**
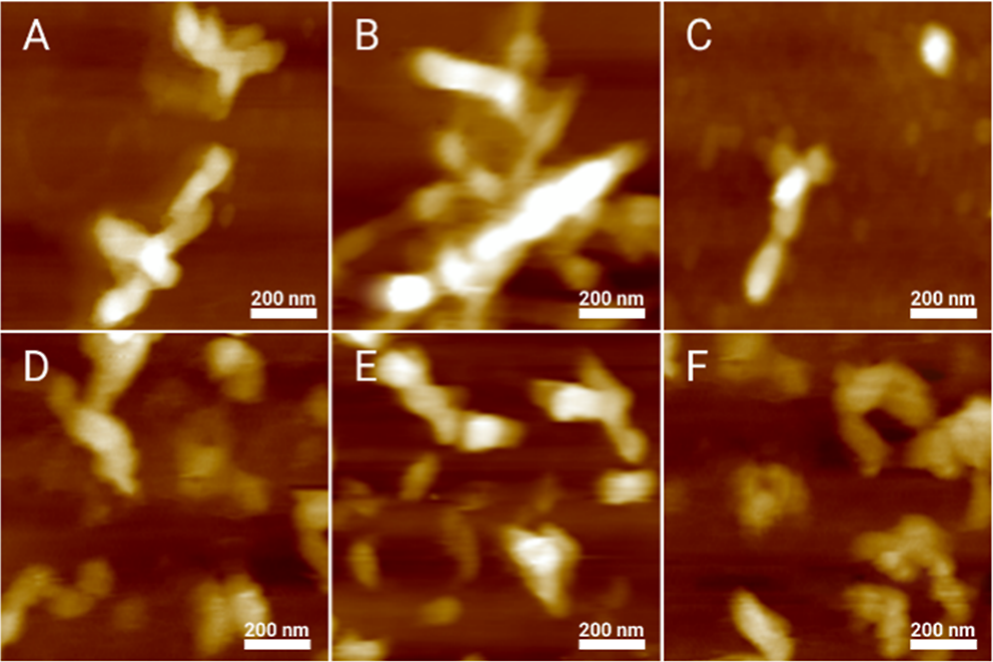
Insulin aggregation in the presence of LUVs with different concentrations of PS yields morphologically similar aggregates. AFM images of insulin aggregates formed in the presence of LUVs of PC/PE/PS (40:40:20) (A), PC/PE/PS (30:40:30) (B), PC/PE/PS (20:40:40) (C), and PC/PE/PS (30:30:40) (D), as well as in the lipid-free environment (E,F).

We utilized AFM to examine the morphology of insulin aggregates grown in the presence of LUVs with different concentrations of PS. We observed fibril-like structures in all analyzed samples. These fibrils had 4–10 nm in height and stretched for 200–500 nm in length, Figures 2, S1. Therefore, we can conclude that the concentration of PS has very little, if any, effect on the morphology of insulin aggregates.

**Figure 3:**
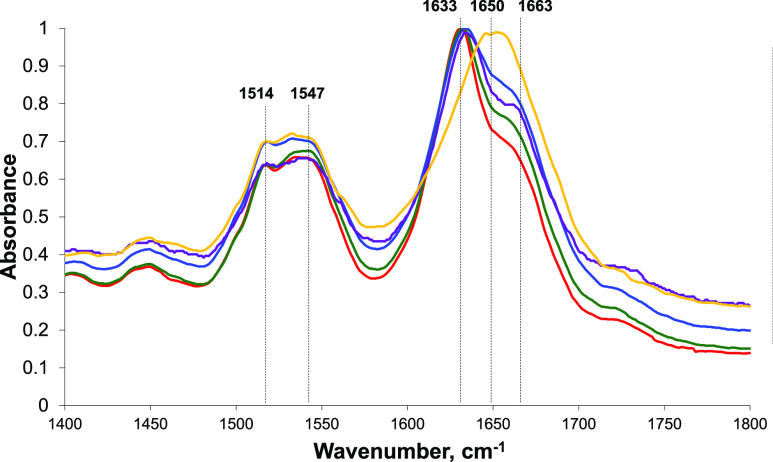
Concentration of PS uniquely alters the secondary structure of insulin aggregates. Average IR spectra of insulin (Ins) fibrils grown in the lipid-free environment (red), insulin in the presence of LUVs of PC/PE/PS (40:40:20) (yellow), PC/PE/PS (30:40:30) (green), PC/PE/PS (20:40:40) (blue), and PC/PE/PS (30:30:40) (purple).

Next, we used IR spectroscopy to examine the secondary structure of insulin fibrils grown in the lipid-free environment as well as protein aggregates formed in the presence of LUVs with different concentrations of PS. IR spectra acquired from Ins fibrils possess both amide I (1630–1700 cm^–1^) and II (1500–1550 cm^–1^) vibrations. The amide I band is centered around 1630 cm^–1^, which indicates the dominance of parallel β-sheets in the secondary structure of Ins fibrils. We also observed a small shoulder at 1663 cm^–1^, which indicates the presence of some unordered protein in the analyzed sample. IR spectra acquired from insulin aggregates grown in the presence of LUVs with PC/PE/PS (30:40:30), PC/PE/PS (20:40:40), and PC/PE/PS (30:30:40) also exhibit an intense band around 1633 cm^–1^ and a shoulder at ∼1663 cm^–1^, which suggests that their secondary structures are dominated by a parallel β-sheet with some unordered protein present, Figure 3. It should be noted that we observed small variations in the position of parallel β-sheet vibration in the analyzed samples. Specifically, in the spectra acquired from insulin aggregates grown in the presence of PC/PE/PS (30:40:30), this vibrational band was centered at 1630 cm^–1^, whereas in the spectra acquired from both Ins/PC/PE/PS (20:40:40) and Ins/PC/PE/PS (30:30:40), this vibration was slightly red-shifted and appeared at ∼1635 cm^–1^, Figure 3. This observation suggests about small changes in the secondary structure of insulin aggregates grown in the presence of LUVs with 40% PS compared to those formed in the lipid-free environment and in the presence of LUVs with 30% PS. It should be also noted that the intensity of the 1663 cm^–1^ band was slightly different in the spectra acquired from Ins, Ins/PC/PE/PS (30:40:30), Ins/PC/PE/PS (20:40:40), and Ins/PC/PE/PS (30:30:40). This observation indicates that these samples had slightly different amounts of unordered protein secondary structures. IR-based analysis of Ins/PC/PE/PS (40:40:20) aggregates revealed major differences in their secondary structure compared to the above-discussed insulin aggregates. Specifically, we found that the amide I band in the spectra acquired form Ins/PC/PE/PS (40:40:20) was shifted to ∼1650 cm^–1^, which suggests that their structure is dominated by unordered protein with some parallel β-sheets present. It should be noted that conventional IR spectroscopy probes the bulk volume of analyzed samples. Since we observed no protein aggregation in the presence of PC/PE/PS (40:40:20) (Figure 1), one may expect that the predominance of an unordered protein signal in the IR spectrum of this sample could be caused by the abundance of unaggregated insulin in Ins/PC/PE/PS (40:40:20).

**Figure 4:**
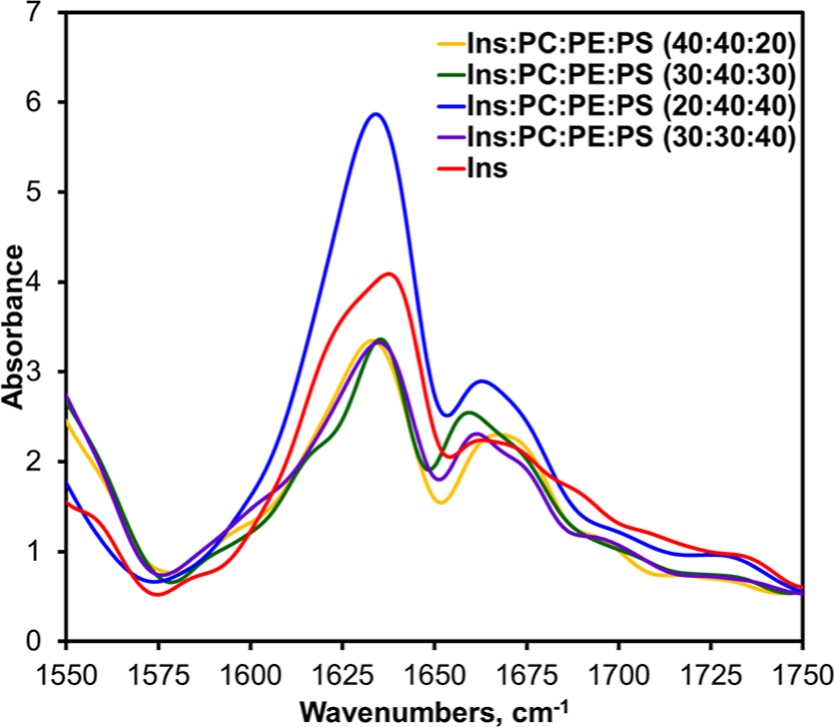
AFM-IR spectra acquired from insulin (Ins) fibrils grown in the lipid-free environment (red), insulin in the presence of LUVs of PC/PE/PS (40:40:20) (yellow), PC/PE/PS (30:40:30) (green), PC/PE/PS (20:40:40) (blue), and PC/PE/PS (30:30:40) (purple).

We also utilized nano-infrared spectroscopy to examine the secondary structure of individual amyloid fibrils formed in the presence of different concentrations of PS, Figure 4. In nano-IR, also known as atomic force microscopy infrared (AFM-IR) spectroscopy, a metallized scanning probe is placed directly on the protein aggregate.^20,21,52^ Next, a pulsed tunable IR laser is used to illuminate the sample, which causes thermal expansion in the protein aggregates.^24,53^ The expansions are recorded by the scanning probe and converted to the IR spectra.^13,15,22^

**Figure 5:**
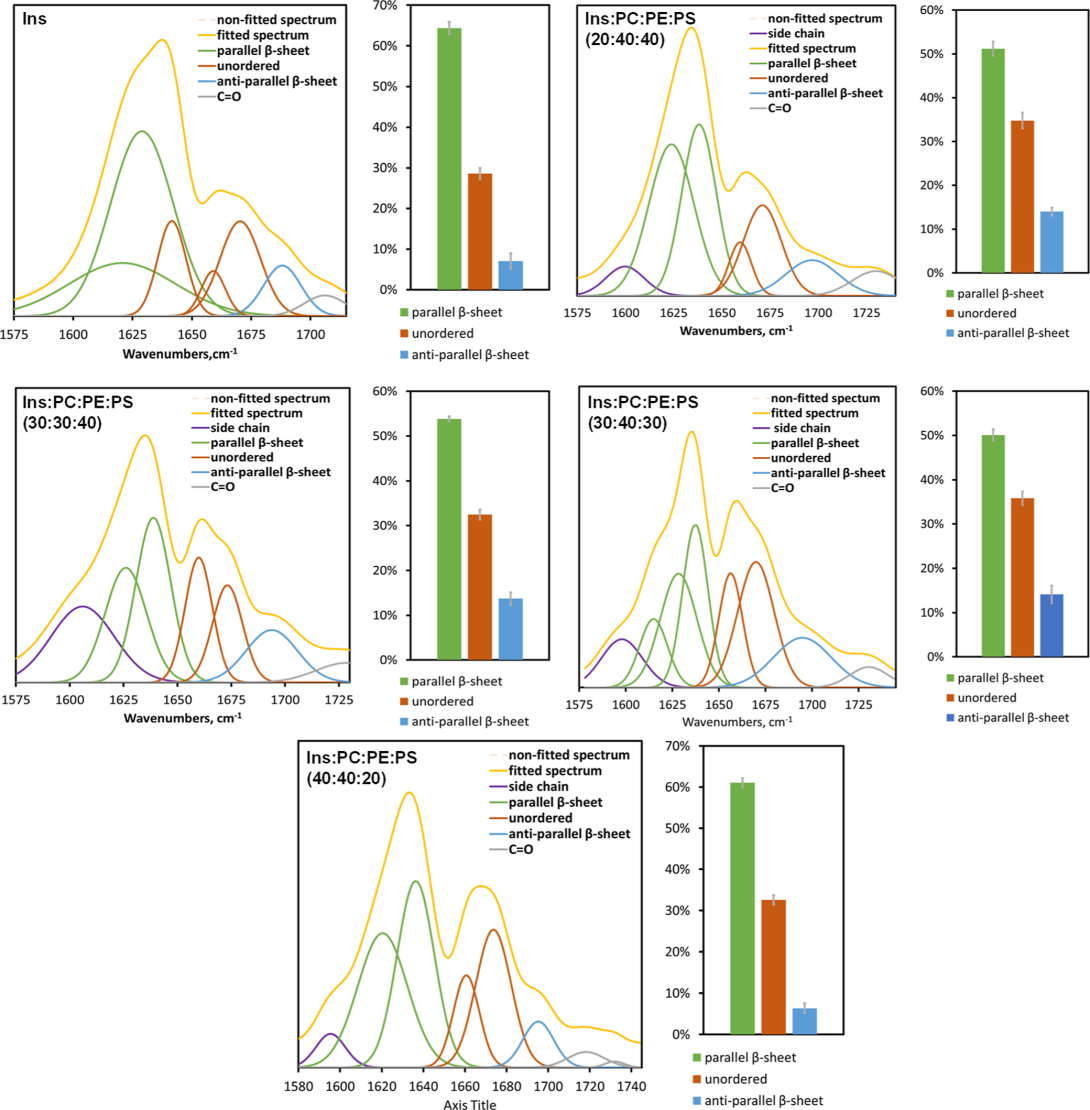
Fitted amide I of AFM-IR spectra acquired from insulin aggregates formed in the lipid-free environment (Ins) and in the presence of PC/PE/PS (20:40:40), PC/PE/PS (30:30:40), PC/PE/PS (30:40:30), and PC/PE/PS (40:40:20) together with the histograms that show distribution of parallel β-sheet, unordered protein, and antiparallel β-sheet in these aggregates.

We found that protein aggregates observed in all analyzed samples exhibited similar AFM-IR spectra with peaks at 1635 and 1660 cm^–1^ in the amide I region, Figure 4. We fitted the amide I band in the acquired AFM-IR spectra to determine the distribution of protein secondary structures, Figure 5. Our results showed that Ins fibrils possessed a higher amount of parallel β-sheet (∼65%) compared to Ins/PC/PE/PS (20:40:40), Ins/PC/PE/PS (30:30:40), Ins/PC/PE/PS (30:40:30), and Ins/PC/PE/PS (40:40:20). We also found that Ins/PC/PE/PS (20:40:40), Ins/PC/PE/PS (30:30:40), and Ins/PC/PE/PS (30:40:30) had very similar secondary structures compared to Ins and Ins/PC/PE/PS (40:40:20). Specifically, Ins and Ins/PC/PE/PS (40:40:20) fibrils had very low amount of anti-parallel β-sheets (∼7%) compared to Ins/PC/PE/PS (20:40:40), Ins/PC/PE/PS (30:30:40), and Ins/PC/PE/PS (30:40:30) (∼14%). Based on these results, we can conclude that Ins/PC/PE/PS (20:40:40), Ins/PC/PE/PS (30:30:40), and Ins/PC/PE/PS (30:40:30) shared a very similar secondary structure, which was different from Ins and Ins/PC/PE/PS (40:40:20). In turn, the secondary structure of Ins/PC/PE/PS (40:40:20) was slightly different from the secondary structure of Ins fibrils. Based on these results, we can conclude that an increase in the concentration of PS from 20 to 30% in LUVs results in the change in the secondary structure of insulin aggregates.

### Toxicity of Insulin Aggregates Grown in the Presence of Different Concentrations of PS

The question to ask is whether the above-discussed changes in the secondary structure of the insulin aggregates grown at the different concentrations of PS have any biological significance. To answer this question, we evaluated the toxicity of insulin aggregates grown in the presence of 20, 30, and 40% PS using LDH assay. We also investigated the extent to which these protein aggregates exert reactive oxygen species (ROS) and alter mitochondrial activity in rat midbrain N27 cells.

**Figure 6:**
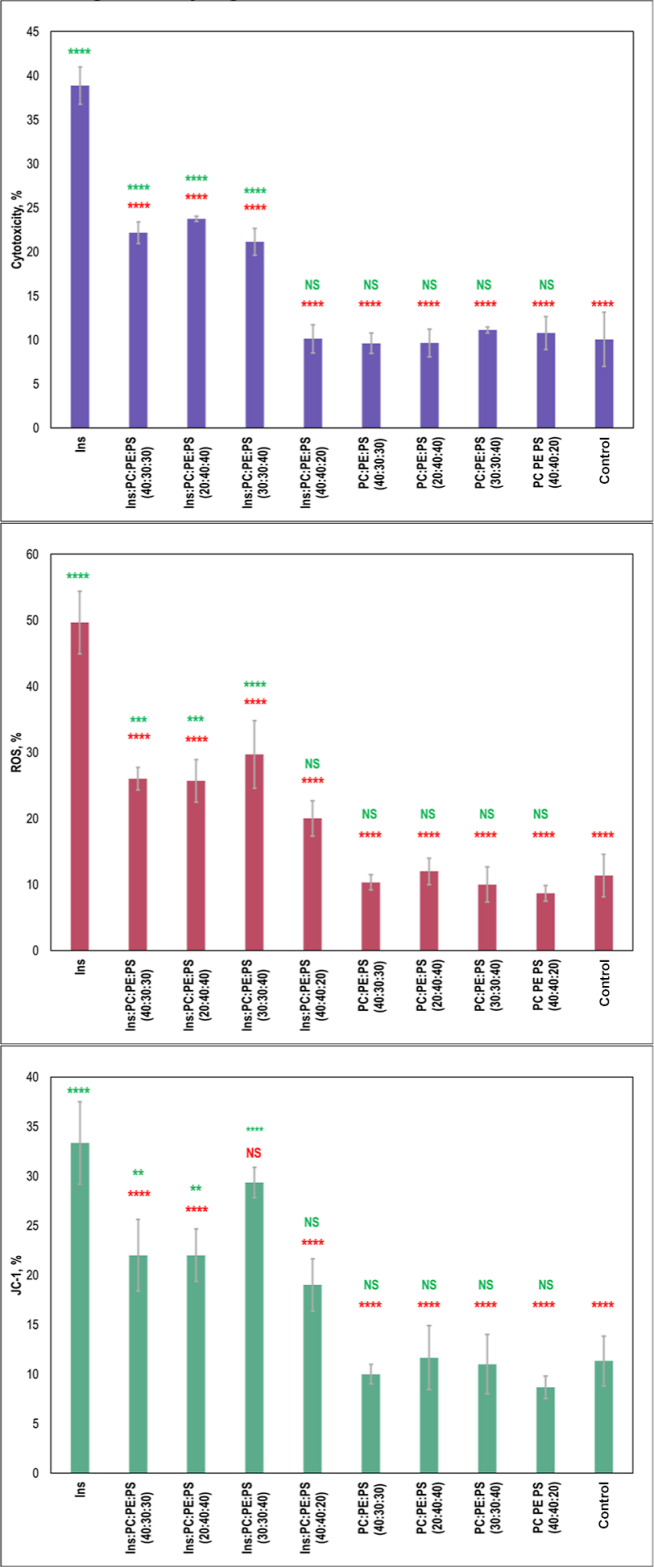
Histograms of LDH (top), ROS (middle), and JC-1 (bottom) toxicity assays of Ins, insulin aggregates grown in the presence of LUVs of PC/PE/PS (40:40:20), PC/PE/PS (30:40:30), PC/PE/PS (20:40:40), and PC/PE/PS (30:30:40), as well as in lipid mixtures themselves. Red asterisks (*) show a significant level of differences between Ins and insulin aggregates grown in the presence of lipids, as well as between lipid samples and the control. Green asterisks (*) show a significant level of differences between the control and insulin aggregates grown in the presence of lipids, as well as between lipid samples themselves. NS is a nonsignificant difference, and *P ≤ 0.05, **P ≤ 0.01, ***P ≤ 0.001, and ****P ≤ 0.0001. One-way ANOVA shows significant differences for all testing groups. Tukey’s HSD post hoc was performed for multiple comparison procedures, and the statistical test showed the following difference between tested groups.

Our results showed that Ins/PC/PE/PS (40:40:20) did not exert any significant cell toxicity compared to the control and lipids themselves, Figure 6. However, Ins/PC/PE/PS (30:40:30) exerted significantly higher cell toxicity compared to Ins/PC/PE/PS (40:40:20), as shown in Figure 6. We also found that the toxicities of Ins/PC/PE/PS (20:40:40) and Ins/PC/PE/PS (30:30:40) were similar to the toxicity of Ins/PC/PE/PS (30:40:30) fibrils. These results demonstrated that an increase in the concentration of PS from 20 to 30% drastically increased the toxicity of insulin aggregates that were formed in the presence of the lipid mixtures, Figure 6. However, we did not observe significant changes in toxicity between 20 and 30%. These results demonstrate that exceeding 20% PS in the plasma membrane could be considered as a critical value that initiates the formation of toxic aggregates that otherwise would not be formed. Finally, it is important to emphasize that Ins fibrils themselves exerted significantly higher cell toxicity compared to all other protein aggregates grown in the presence of lipids, which is consistent with our previously reported results.

Similar differences between insulin fibrils grown in the lipid-free environment and in the presence of lipid mixtures were observed for exerted ROS levels, Figure 6. Specifically, we found that Ins fibrils exerted significantly higher levels of ROS compared to all samples that were grown in the presence of lipids. We also found that Ins/PC/PE/PS (40:40:20) exerted some ROS stress; however, its magnitude was lower compared to the levels of ROS observed for Ins/PC/PE/PS (30:40:30), Ins/PC/PE/PS (20:40:40), and Ins/PC/PE/PS (30:30:40). It should be noted that lipid mixtures themselves did not exert any significant levels of ROS to rat midbrain N27 cells.

Our results showed that Ins/PC/PE/PS (40:40:20) did not exert any significant cell toxicity compared to the control and lipids themselves, Figure 6. However, Ins/PC/PE/PS (30:40:30) exerted significantly higher cell toxicity compared to Ins/PC/PE/PS (40:40:20). We also found that the toxicities of Ins/PC/PE/PS (20:40:40) and Ins/PC/PE/PS (30:30:40) were similar to the toxicity of Ins/PC/PE/PS (30:40:30) fibrils. These results demonstrated that an increase in the concentration of PS from 20 to 30% drastically increased the toxicity of insulin aggregates that were formed in the presence of the lipid mixtures. However, we did not observe significant changes in toxicity between 20 and 30%. These results demonstrate that exceeding 20% PS in the plasma membrane could be considered as a critical value that initiates the formation of toxic aggregates that otherwise would not be formed. Finally, it is important to emphasize that Ins fibrils themselves exerted significantly higher cell toxicity compared to all other protein aggregates grown in the presence of lipids, which is consistent with our previously reported results.

We utilized JC-1 assay to determine the extent to which insulin aggregates impaired mitochondrial activity in mouse midbrain N27 cells, Figure 6. We found that Ins/PC/PE/PS (40:40:20) caused substantial mitochondrial dysfunction that was comparable to the level of damage exerted by Ins/PC/PE/PS (30:40:30) and Ins/PC/PE/PS (20:40:40) aggregates. Thus, although Ins/PC/PE/PS (40:40:20) was found to be insignificantly toxic, these aggregates damage mitochondrial activities of these cells. We also found that insulin aggregates that were formed in the presence of Ins/PC/PE/PS (30:30:40) exerted higher mitochondrial dysfunction than any other protein aggregates formed in the presence of lipid mixtures. Furthermore, the level of mitochondrial dysfunction caused by Ins/PC/PE/PS (30:30:40) was comparable to the levels exerted by Ins. These results suggest that the ratio between PC and PE could play some role in the toxicity of insulin aggregates formed in the presence of these zwitterionic lipids.

## Discussion

Our results suggest that elevated concentrations of PS in the plasma membrane can be a trigger of abrupt aggregation of misfolded proteins. At physiological concentrations of PS (≤20%), PC and PE strongly inhibit the formation of fibrils. Therefore, plasma and organelle membranes are dominated by such zwitterionic lipids. However, an increase in the concentration of PS above 20% results in the acceleration of protein aggregation. These results are in good agreement with the previously reported by our and other research group effect of anionic lipids.

AFM imaging revealed that at 20, 30, and 40% PS, morphologically similar aggregates were observed. However, the secondary structure of these aggregates was substantially different. Specifically, at 20% PS, we observed the formation of insulin aggregates that had a mixture of unordered protein and parallel β-sheet present, whereas at the higher concentrations of PS (30 and 40% PS), insulin fibrils possessed primarily parallel β-sheet present secondary structures. Our results also showed that insulin fibrils that were grown in the presence of different concentrations of PS exhibit similar cell toxicity that was lower compared to the toxicity exerted by insulin fibrils grown in the lipid-free environment. Our results also show that insulin fibrils that were grown in the presence of different concentrations of PS exert their toxicity enhancing ROS levels simultaneously causing mitochondrial disfunction.

These findings suggest that apoptotic cells with a high concentration of PS in their plasma membranes could trigger aggregation of amyloidogenic proteins if not eliminated by macrophages. It is important to emphasize that protein aggregation in such cases will be triggered by the external rather than intercellular factors. This hypothesis, however, does not exclude intercellular protein aggregation, which could take place in endosomes and multivesicular bodies.^54−57^ For instance, Almeida and co-workers demonstrated that amyloid β peptide can accumulate in the multivesicular bodies, which results in fibril formation and cell death.^54^

## Conclusions

Our results demonstrate that an increase in the concentration of anionic PS above 20% in the plasma membrane triggers the abrupt protein aggregation. This results in the formation of morphologically similar protein aggregates that have distinctly different secondary structures. We also found that insulin aggregates grown in the presence of 20, 30, and 40% PS exhibited similar cell toxicity, which is significantly lower than the toxicity of insulin aggregates grown in the absence of lipids. These results demonstrate an important role of anionic lipids in the protein aggregation. Our results also suggest that a deceleration in the efficiency of clearance of apoptotic and pre-apoptotic cells by macrophages can be the underlying cause of the the abrupt protein aggregation that will take place on the plasma membranes of such misfunctioning cells. Specifically, high concentrations of PS on their surfaces can trigger aggregation of amyloidogenic proteins such as insulin, lysozyme, and serum amyloid A that are present in the extracellular space.

## Experimental Section

### Materials

Bovine insulin was purchased from Sigma-Aldrich (St. Louis, MO, USA), and 1,2-dimyristoyl-*sn*-glycero-3-phospho-l-serine (DMPS or PS), 1,2-dimyristoyl-*sn*-glycero-3-phosphocholine (DMPC or PC), and 1,2-dimyristoyl-*sn*-glycero-3-phosphoethanolamine (DMPE or PE) were purchased from Avanti (Alabaster, AL, USA).

### Liposome Preparation

We mixed PC, PE, and PS in the following molar ratios 40:40:20, 30:40:30, 20:40:40, and 30:30:40 in glass vials. Next, lipids were dissolved in chloroform until powders were fully dissolved. After chloroform was fully evaporated under dry nitrogen, the lipid film was dissolved in phosphate buffered saline (PBS), pH 7.4. Once lipids were fully dissolved, solutions were heated to ∼50 °C in a water bath for 30 min. After that, lipid solutions were immersed into liquid nitrogen for 3–5 min. The procedure was repeated 8–10 times. This procedure was repeated 10 times to enable the formation of homogeneous liposomes. Next, lipid solutions were subjected to fine reshaping on the LUVs using an extruder equipped with a 100 nm membrane (Avanti, Alabaster, AL, USA). We used dynamic light scattering to verify that the size of the LUVs was within 100 ± 10 nm, Figure S2. The question to ask is to what extent all LUVs in the same sample had exactly the same percentages of all PC, PE, and PS. Although we have no direct experimental data that demonstrate the uniformity of such lipid compositions in LUVs, our kinetic studies of insulin aggregation in the presence of LUVs with different concentrations of PS indirectly support our presumption that LUVs present in the same sample had very similar, if not identical, ratios of PC, PE, and PS (Figure 1 and Table S1). One may expect that in the case of absence of expected homogeneity of LUVs, much greater variability in the aggregation kinetics could be expected because PS itself strongly accelerated insulin aggregation, whereas PC and PE, on the other hand, fully inhibited protein aggregation.^58^

### Insulin Aggregation

In the lipid-free environment, 400 μM of insulin was dissolved in PBS, and the solution pH was adjusted to pH 3.0 using 10–15 μL of 1 M HCl. In parallel, 400 μM of insulin was mixed with an equivalent concentration of the corresponding lipid mixture. After that, the solution pH was adjusted to pH 3.0 by adding 10–15 μL of 1 M HCl. Samples were placed into a 96-well plate that was kept at 37 °C for 70 h under 510 rpm agitation in the plate reader (Tecan, Männedorf, Switzerland).

### Kinetic Measurements

To measure the rates of insulin aggregation, thioflavin T (ThT) fluorescence assay was used. We mixed samples with 2 mM of ThT solution and incubated them under the same experimental conditions as described above (37 °C for 70 h under 510 rpm agitation). Fluorescence measurements were taken in the plate reader (Tecan, Männedorf, Switzerland) every 10 min with excitation at 450 nm. The emission signal was recorded at 488 nm.

### AFM Imaging

AFM images were acquired on a Nano-IR3 system (Bruker, Santa Barbara, CA, USA) equipped with contact-mode AFM scanning probes (ContGB-G AFM probe, NanoAndMore, Watsonville, CA, USA). For the imaging, an aliquot of protein solution was placed onto a silicon water. After the solution was exposed on the water surface for 2–3 min, it was removed. The water was dried under a flow of nitrogen.

### Attenuated Total Reflectance Fourier-Transform Infrared Spectroscopy

After 70 h of sample incubation, an aliquot of protein solution was placed onto the ATR crystal and dried at room temperature. Spectra were measured using a Spectrum 100 FTIR spectrometer (Perkinelmer, Waltham, MA, USA). Three spectra were collected from each sample.

### Cell Toxicity Assays

Rat midbrain N27 cells were grown in RPMI 1640 Medium (Thermo Fisher Scientific, Waltham, MA, USA) with 10% fetal bovine serum (FBS) (Invitrogen, Waltham, MA, USA) in 96-well plate (5000 cells per well) at 37 °C under 5% CO_2_. After 24 h, the cells were found to fully adhere to the wells, reaching approximately 70% confluency. Rat neuronal N27 cells divide approximately once in 12 h. Thus, after 24 h of cell incubation at 37 °C under 5% CO_2_, each well contained approximately 10,000 cells. The cell count and viability were verified by Trypan Blue dye that confirmed the number of cells and their viability in the range of 98–99%. Next, 100 μL of the cell culture was replaced with 100 μL RPMI 1640 Medium with 5% FBS containing protein aggregates formed after 70 h of sample incubation at 37 °C. After 48 h of incubation, lactate dehydrogenase assay was performed on the cell medium using CytoTox 96 non-radioactive cytotoxicity assay (G1781, Promega, Madison, WI, USA). Absorption measurements were made in plate reader (Tecan, Männedorf, Switzerland) at 490 nm. Every well was measured 25 times in different locations.

In parallel, ROS assay was performed on the same cell culture. For this, ROS reagent (C10422, Invitrogen, Waltham, MA, USA) was added to reach the final concentration of 5 μM; cells were incubated at 37 °C under 5% CO_2_ for 30 min. After the supernatant was removed, cells were washed with PBS and resuspended in 200 μL of PBS in the flow cytometry tubes. Sample measurements were made in an LSRII BD flow cytometer (BD, San Jose, CA, USA) using red channel (λ = 633 nm), Figure S3. Percentages of ROS cells were determined using LSRII software.

For JC-1 staining, 1 μL of JC-1 reagent (M34152A, Invitrogen, Waltham, MA, USA) was added to cells and incubated at 37 °C under 5% CO_2_ for 30 min. After the supernatant was removed, cells were washed with PBS and resuspended in 200 μL of PBS in the flow cytometry tubes. Sample measurements were made in an LSRII BD flow cytometer (BD, San Jose, CA, USA) using a red channel (λ = 633 nm), Figure S4. Percentages of ROS cells were determined using LSRII BD software.

## Supporting Information Available

DLS results of LUV analyses and histograms of height profiles of amyloid aggregates; and flow cytometry plots (PDF)

## References

[ref1] Chiti F.; Dobson C. M. Protein Misfolding, Amyloid Formation, and Human Disease: A Summary of Progress Over the Last Decade. Annu. Rev. Biochem. 2017, 86, 27–68. 10.1146/annurev-biochem-061516-045115. 28498720

[ref2] Knowles T. P.; Vendruscolo M.; Dobson C. M. The amyloid state and its association with protein misfolding diseases. Nat. Rev. 2014, 15, 384–396. 10.1038/nrm3810. 24854788

[ref3] Chen S. W.; Drakulic S.; Deas E.; Ouberai M.; Aprile F. A.; Arranz R.; Ness S.; Roodveldt C.; Guilliams T.; De-Genst E. J.; Klenerman D.; Wood N. W.; Knowles T. P.; Alfonso C.; Rivas G.; Abramov A. Y.; Valpuesta J. M.; Dobson C. M.; Cremades N. Structural characterization of toxic oligomers that are kinetically trapped during alpha-synuclein fibril formation. Proc. Natl. Acad. Sci. U.S.A. 2015, 112, E1994–E2003. 10.1073/pnas.1421204112. 25855634 PMC4413268

[ref4] Sandberg A.; Luheshi L. M.; Sollvander S.; Pereira de Barros T.; Macao B.; Knowles T. P.; Biverstal H.; Lendel C.; Ekholm-Petterson F.; Dubnovitsky A.; Lannfelt L.; Dobson C. M.; Hard T. Stabilization of neurotoxic Alzheimer amyloid-beta oligomers by protein engineering. Proc. Natl. Acad. Sci. U.S.A. 2010, 107, 15595–15600. 10.1073/pnas.1001740107. 20713699 PMC2932621

[ref5] Zhaliazka K.; Kurouski D. Nanoscale Characterization of Parallel and Antiparallel beta-Sheet Amyloid Beta 1-42 Aggregates. ACS Chem. Neurosci. 2022, 13, 2813–2820. 10.1021/acschemneuro.2c00180. 36122250 PMC10405294

[ref6] Kurouski D.; Lu X.; Popova L.; Wan W.; Shanmugasundaram M.; Stubbs G.; Dukor R. K.; Lednev I. K.; Nafie L. A. Is supramolecular filament chirality the underlying cause of major morphology differences in amyloid fibrils?. J. Am. Chem. Soc. 2014, 136, 2302–2312. 10.1021/ja407583r. 24484302 PMC3968177

[ref7] Ghosh U.; Yau W. M.; Collinge J.; Tycko R. Structural differences in amyloid-beta fibrils from brains of nondemented elderly individuals and Alzheimer’s disease patients. Proc. Natl. Acad. Sci. U.S.A. 2021, 118, e2111863118 10.1073/pnas.2111863118. 34725161 PMC8609303

[ref8] Guerrero-Ferreira R.; Taylor N. M.; Mona D.; Ringler P.; Lauer M. E.; Riek R.; Britschgi M.; Stahlberg H. Cryo-EM structure of alpha-synuclein fibrils. Elife 2018, 7, e36402 10.7554/elife.36402. 29969391 PMC6092118

[ref9] Kollmer M.; Close W.; Funk L.; Rasmussen J.; Bsoul A.; Schierhorn A.; Schmidt M.; Sigurdson C. J.; Jucker M.; Fandrich M. Cryo-EM structure and polymorphism of Aβ amyloid fibrils purified from Alzheimer’s brain tissue. Nat. Commun. 2019, 10, 4760 10.1038/s41467-019-12683-8. 31664019 PMC6820800

[ref10] Rizevsky S.; Matveyenka M.; Kurouski D. Nanoscale Structural Analysis of a Lipid-Driven Aggregation of Insulin. J. Phys. Chem. Lett. 2022, 13, 2467–2473. 10.1021/acs.jpclett.1c04012. 35266717 PMC9169669

[ref11] Paravastu A. K.; Qahwash I.; Leapman R. D.; Meredith S. C.; Tycko R. Seeded growth of beta-amyloid fibrils from Alzheimer’s brain-derived fibrils produces a distinct fibril structure. Proc. Natl. Acad. Sci. U.S.A. 2009, 106, 7443–7448. 10.1073/pnas.0812033106. 19376973 PMC2678625

[ref12] Rizevsky S.; Kurouski D. Nanoscale Structural Organization of Insulin Fibril Polymorphs Revealed by Atomic Force Microscopy-Infrared Spectroscopy (AFM-IR). Chembiochem 2020, 21, 481–485. 10.1002/cbic.201900394. 31299124

[ref13] Ruggeri F. S.; Charmet J.; Kartanas T.; Peter Q.; Chia S.; Habchi J.; Dobson C. M.; Vendruscolo M.; Knowles T. P. J. Microfluidic deposition for resolving single-molecule protein architecture and heterogeneity. Nat. Commun. 2018, 9, 3890 10.1038/s41467-018-06345-4. 30250131 PMC6155325

[ref14] Ruggeri F. S.; Flagmeier P.; Kumita J. R.; Meisl G.; Chirgadze D. Y.; Bongiovanni M. N.; Knowles T. P. J.; Dobson C. M. The Influence of Pathogenic Mutations in alpha-Synuclein on Biophysical and Structural Characteristics of Amyloid Fibrils. ACS Nano 2020, 14, 5213–5222. 10.1021/acsnano.9b09676. 32159944

[ref15] Ruggeri F. S.; Longo G.; Faggiano S.; Lipiec E.; Pastore A.; Dietler G. Infrared nanospectroscopy characterization of oligomeric and fibrillar aggregates during amyloid formation. Nat. Commun. 2015, 6, 7831 10.1038/ncomms8831. 26215704 PMC4525161

[ref16] Zhou L.; Kurouski D. Structural Characterization of Individual alpha-Synuclein Oligomers Formed at Different Stages of Protein Aggregation by Atomic Force Microscopy-Infrared Spectroscopy. Anal. Chem. 2020, 92, 6806–6810. 10.1021/acs.analchem.0c00593. 32347706

[ref17] Dou T.; Li Z.; Zhang J.; Evilevitch A.; Kurouski D. Nanoscale Structural Characterization of Individual Viral Particles Using Atomic Force Microscopy Infrared Spectroscopy (AFM-IR) and Tip-Enhanced Raman Spectroscopy (TERS). Anal. Chem. 2020, 92, 11297–11304. 10.1021/acs.analchem.0c01971. 32683857

[ref18] Rizevsky S.; Zhaliazka K.; Dou T.; Matveyenka M.; Kurouski D. Characterization of Substrates and Surface-Enhancement in Atomic Force Microscopy Infrared Analysis of Amyloid Aggregates. J. Phys. Chem. C 2022, 126, 4157–4162. 10.1021/acs.jpcc.1c09643. PMC9205157 35719853

[ref19] Dazzi A.; Glotin F.; Carminati R. Theory of infrared nanospectroscopy by photothermal induced resonance. J. Appl. Phys. 2010, 107, 124519 10.1063/1.3429214.

[ref20] Dazzi A.; Prater C. B. AFM-IR: Technology and Applications in Nanoscale Infrared Spectroscopy and Chemical Imaging. Chem. Rev. 2017, 117, 5146–5173. 10.1021/acs.chemrev.6b00448. 27958707

[ref21] Kurouski D.; Dazzi A.; Zenobi R.; Centrone A. Infrared and Raman chemical imaging and spectroscopy at the nanoscale. Chem. Soc. Rev. 2020, 49, 3315–3347. 10.1039/c8cs00916c. 32424384 PMC7675782

[ref22] Ruggeri F. S.; Benedetti F.; Knowles T. P. J.; Lashuel H. A.; Sekatskii S.; Dietler G. Identification and nanomechanical characterization of the fundamental single-strand protofilaments of amyloid alpha-synuclein fibrils. Proc. Natl. Acad. Sci. U.S.A. 2018, 115, 7230–7235. 10.1073/pnas.1721220115. 29941606 PMC6048494

[ref23] Ruggeri F. S.; Vieweg S.; Cendrowska U.; Longo G.; Chiki A.; Lashuel H. A.; Dietler G. Nanoscale studies link amyloid maturity with polyglutamine diseases onset. Sci. Rep. 2016, 6, 31155 10.1038/srep31155. 27499269 PMC4976327

[ref24] Ramer G.; Ruggeri F. S.; Levin A.; Knowles T. P. J.; Centrone A. Determination of Polypeptide Conformation with Nanoscale Resolution in Water. ACS Nano 2018, 12, 6612–6619. 10.1021/acsnano.8b01425. 29932670 PMC11404133

[ref25] Farber C.; Li J.; Hager E.; Chemelewski R.; Mullet J.; Rogachev A. Y.; Kurouski D. Complementarity of Raman and Infrared Spectroscopy for Structural Characterization of Plant Epicuticular Waxes. ACS Omega 2019, 4, 3700–3707. 10.1021/acsomega.8b03675.

[ref26] Farber C.; Wang R.; Chemelewski R.; Mullet J.; Kurouski D. Nanoscale Structural Organization of Plant Epicuticular Wax Probed by Atomic Force Microscope Infrared Spectroscopy. Anal. Chem. 2019, 91, 2472–2479. 10.1021/acs.analchem.8b05294. 30624904

[ref27] Wieland K.; Ramer G.; Weiss V. U.; Allmaier G.; Lendl B.; Centrone A. Nanoscale chemical imaging of individual chemotherapeutic cytarabine-loaded liposomal nanocarriers. Nano Res. 2019, 12, 197–203. 10.1007/s12274-018-2202-x. PMC6604632 31275527

[ref28] Perez-Guaita D.; Kochan K.; Batty M.; Doerig C.; Garcia-Bustos J.; Espinoza S.; McNaughton D.; Heraud P.; Wood B. R. Multispectral Atomic Force Microscopy-Infrared Nano-Imaging of Malaria Infected Red Blood Cells. Anal. Chem. 2018, 90, 3140–3148. 10.1021/acs.analchem.7b04318. 29327915

[ref29] Dazzi A. PhotoThermal Induced Resonance. Application to Infrared Spectromicroscopy Volz S., Ed.; Thermal Nanosystems and Nanomaterials; Springer Berlin, 2009; Vol. 118, pp 469–503.

[ref30] Dazzi A.; Prazeres R.; Glotin F.; Ortega J. M.; Al-Sawaftah M.; de Frutos M. Chemical mapping of the distribution of viruses into infected bacteria with a photothermal method. Ultramicroscopy 2008, 108, 635–641. 10.1016/j.ultramic.2007.10.008. 18037564

[ref31] Mayet C.; Deniset-Besseau A.; Prazeres R.; Ortega J. M.; Dazzi A. Analysis of bacterial polyhydroxybutyrate production by multimodal nanoimaging. Biotechnol. Adv. 2013, 31, 369–374. 10.1016/j.biotechadv.2012.05.003. 22634017

[ref32] Kochan K.; Perez-Guaita D.; Pissang J.; Jiang J. H.; Peleg A. Y.; McNaughton D.; Heraud P.; Wood B. R. In vivo atomic force microscopy-infrared spectroscopy of bacteria. J. R. Soc., Interface 2018, 15, 20180115 10.1098/rsif.2018.0115. 29593091 PMC5908543

[ref33] Kurouski D. Advances of tip-enhanced Raman spectroscopy (TERS) in electrochemistry, biochemistry, and surface science. Vib. Spectrosc. 2017, 91, 3–15. 10.1016/j.vibspec.2016.06.004.

[ref34] Verma P. Tip-Enhanced Raman Spectroscopy: Technique and Recent Advances. Chem. Rev. 2017, 117, 6447–6466. 10.1021/acs.chemrev.6b00821. 28459149

[ref35] Kurouski D.; Deckert-Gaudig T.; Deckert V.; Lednev I. K. Structure and composition of insulin fibril surfaces probed by TERS. J. Am. Chem. Soc. 2012, 134, 13323–13329. 10.1021/ja303263y. 22813355 PMC3426279

[ref36] Kurouski D.; Deckert-Gaudig T.; Deckert V.; Lednev I. K. Surface characterization of insulin protofilaments and fibril polymorphs using tip-enhanced Raman spectroscopy (TERS). Biophys. J. 2014, 106, 263–271. 10.1016/j.bpj.2013.10.040. 24411258 PMC3907223

[ref37] Bonhommeau S.; Talaga D.; Hunel J.; Cullin C.; Lecomte S. Tip-Enhanced Raman Spectroscopy to Distinguish Toxic Oligomers from Aβ_1–42_ Fibrils at the Nanometer Scale. Angew. Chem., Int. Ed. Engl. 2017, 56, 1771–1774. 10.1002/anie.201610399. 28071842

[ref38] Matveyenka M.; Rizevsky S.; Kurouski D. Unsaturation in the Fatty Acids of Phospholipids Drastically Alters the Structure and Toxicity of Insulin Aggregates Grown in Their Presence. J. Phys. Chem. Lett. 2022, 13, 4563–4569. 10.1021/acs.jpclett.2c00559. 35580189 PMC9170185

[ref39] Matveyenka M.; Rizevsky S.; Kurouski D. The degree of unsaturation of fatty acids in phosphatidylserine alters the rate of insulin aggregation and the structure and toxicity of amyloid aggregates. FEBS Lett. 2022, 596, 1424–1433. 10.1002/1873-3468.14369. 35510803 PMC9197964

[ref40] Matveyenka M.; Rizevsky S.; Kurouski D. Length and Unsaturation of Fatty Acids of Phosphatidic Acid Determines the Aggregation Rate of Insulin and Modifies the Structure and Toxicity of Insulin Aggregates. ACS Chem. Neurosci. 2022, 13, 2483–2489. 10.1021/acschemneuro.2c00330. 35930674

[ref41] Matveyenka M.; Rizevsky S.; Kurouski D. Amyloid aggregates exert cell toxicity causing irreversible damages in the endoplasmic reticulum. Biochim. Biophys. Acta, Mol. Basis Dis. 2022, 1868, 166485 10.1016/j.bbadis.2022.166485. 35840040 PMC10424722

[ref42] Matveyenka M.; Rizevsky S.; Pellois J. P.; Kurouski D. Lipids uniquely alter rates of insulin aggregation and lower toxicity of amyloid aggregates. Biochim. Biophys. Acta, Mol. Cell Biol. Lipids 2023, 1868, 159247 10.1016/j.bbalip.2022.159247. 36272517 PMC10401553

[ref43] Matveyenka M.; Zhaliazka K.; Rizevsky S.; Kurouski D. Lipids uniquely alter secondary structure and toxicity of lysozyme aggregates. FASEB J. 2022, 36, e22543 10.1096/fj.202200841r. 36094052 PMC10427241

[ref44] Zhaliazka K.; Rizevsky S.; Matveyenka M.; Serada V.; Kurouski D. Charge of Phospholipids Determines the Rate of Lysozyme Aggregation but Not the Structure and Toxicity of Amyloid Aggregates. J. Phys. Chem. Lett. 2022, 13, 8833–8839. 10.1021/acs.jpclett.2c02126. 36111888 PMC10405293

[ref45] Alza N. P.; Iglesias Gonzalez P. A.; Conde M. A.; Uranga R. M.; Salvador G. A. Lipids at the Crossroad of alpha-Synuclein Function and Dysfunction: Biological and Pathological Implications. Front. Cell. Neurosci. 2019, 13, 175 10.3389/fncel.2019.00175. 31118888 PMC6504812

[ref46] Galvagnion C. The Role of Lipids Interacting with-Synuclein in the Pathogenesis of Parkinson’s Disease. J. Parkinson’s Dis. 2017, 7, 433–450. 10.3233/jpd-171103. 28671142

[ref47] Galvagnion C.; Brown J. W.; Ouberai M. M.; Flagmeier P.; Vendruscolo M.; Buell A. K.; Sparr E.; Dobson C. M. Chemical properties of lipids strongly affect the kinetics of the membrane-induced aggregation of alpha-synuclein. Proc. Natl. Acad. Sci. U.S.A. 2016, 113, 7065–7070. 10.1073/pnas.1601899113. 27298346 PMC4932957

[ref48] Dou T.; Zhou L.; Kurouski D. Unravelling the Structural Organization of Individual alpha-Synuclein Oligomers Grown in the Presence of Phospholipids. J. Phys. Chem. Lett. 2021, 12, 4407–4414. 10.1021/acs.jpclett.1c00820. 33945282

[ref49] Dou T.; Kurouski D. Phosphatidylcholine and Phosphatidylserine Uniquely Modify the Secondary Structure of alpha-Synuclein Oligomers Formed in Their Presence at the Early Stages of Protein Aggregation. ACS Chem. Neurosci. 2022, 13, 2380–2385. 10.1021/acschemneuro.2c00355. 35904551 PMC10405296

[ref50] Zhang X.; St Clair J. R.; London E.; Raleigh D. P. Islet Amyloid Polypeptide Membrane Interactions: Effects of Membrane Composition. Biochemistry 2017, 56, 376–390. 10.1021/acs.biochem.6b01016. 28054763 PMC5541234

[ref51] Avdulov N. A.; Chochina S. V.; Igbavboa U.; Warden C. S.; Vassiliev A. V.; Wood W. G. Lipid binding to amyloid beta-peptide aggregates: preferential binding of cholesterol as compared with phosphatidylcholine and fatty acids. J. Neurochem. 1997, 69, 1746–1752. 10.1046/j.1471-4159.1997.69041746.x. 9326304

[ref52] Centrone A. Infrared imaging and spectroscopy beyond the diffraction limit. Annu. Rev. Anal. Chem. 2015, 8, 101–126. 10.1146/annurev-anchem-071114-040435. 26001952

[ref53] Katzenmeyer A. M.; Aksyuk V.; Centrone A. Nanoscale infrared spectroscopy: improving the spectral range of the photothermal induced resonance technique. Anal. Chem. 2013, 85, 1972–1979. 10.1021/ac303620y. 23363013

[ref54] Almeida C. G.; Takahashi R. H.; Gouras G. K. β-Amyloid Accumulation Impairs Multivesicular Body Sorting by Inhibiting the Ubiquitin-Proteasome System. J. Neurosci. 2006, 26, 4277–4288. 10.1523/jneurosci.5078-05.2006. 16624948 PMC6673997

[ref55] Watt B.; van Niel G.; Fowler D. M.; Hurbain I.; Luk K. C.; Stayrook S. E.; Lemmon M. A.; Raposo G.; Shorter J.; Kelly J. W.; Marks M. S. N-terminal domains elicit formation of functional Pmel17 amyloid fibrils. J. Biol. Chem. 2009, 284, 35543–35555. 10.1074/jbc.m109.047449. 19840945 PMC2790984

[ref56] Willen K.; Edgar J. R.; Hasegawa T.; Tanaka N.; Futter C. E.; Gouras G. K. Aβ accumulation causes MVB enlargement and is modelled by dominant negative VPS4A. Mol. Neurodegener. 2017, 12, 61 10.1186/s13024-017-0203-y. 28835279 PMC5569475

[ref57] Schutzmann M. P.; Hasecke F.; Bachmann S.; Zielinski M.; Hansch S.; Schroder G. F.; Zempel H.; Hoyer W. Endo-lysosomal Aβ concentration and pH trigger formation of Aβ oligomers that potently induce Tau missorting. Nat. Commun. 2021, 12, 4634 10.1038/s41467-021-24900-4. 34330900 PMC8324842

[ref58] Matveyenka M.; Rizevsky S.; Kurouski D. Elucidation of the Effect of Phospholipid Charge on the Rate of Insulin Aggregation and Structure and Toxicity of Amyloid Fibrils. ACS Omega 2023, 8, 12379–12386. 10.1021/acsomega.3c00159. 37033844 PMC10077570

