# Supplementary material for: Concentration of Phosphatidylserine Influence Rates of Insulin Aggregation and Toxicity of Amyloid Aggregates In Vitro
Source: ACS Chem Neurosci. 2023 Jun 6;14(12):2396–404. doi: 10.1021/acschemneuro.3c00277 (PMC10401552; doi:10.1021/acschemneuro.3c00277)
Supplement: Supplementary file 1 — cn3c00277_si_001.pdf [file cn3c00277_si_001.pdf]

The Concentration of Phosphatidylserine Influence Rates of Insulin Aggregation and Toxicity of Amyloid Aggregates *In Vitro*.

Mikhail Matveyenka[1], Kiryl Zhaliazka[1] and Dmitry Kurouski[*1,2]

1. Department of Biochemistry and Biophysics, Texas A&M University, College Station, Texas 77843, United States
2. Department of Biomedical Engineering, Texas A&M University, College Station, Texas, 77843, United States

Corresponding author: dkurouski@tamu.edu

Supporting Information

Table S1. Results of t_{lag} and $t_{1/2}$ for insulin aggregation in the lipid-free environment (Ins), as well as in the presence of LUVs with different ratios of PS.

	Ins	Ins PC:PE:PS (20:40:40)	Ins PC:PE:PS (30:30:40)	Ins PC:PE:PS (30:40:30)
t_{lag}	13,5 h	22,1 h	25,7 h	35,9 h
	12,6 h	24,5 h	27,1 h	36,9 h
	13,9 h	25,8 h	27,3 h	37,3 h
$t_{1/2}$	15,1 h	16,1 h	24,4 h	37,2 h
	14,3 h	18,1 h	25,5 h	38,3 h
	15,8 h	21,2 h	25,5 h	38,5 h
	Ins	Ins PC:PE:PS (20:40:40)	Ins PC:PE:PS (30:30:40)	Ins PC:PE:PS (30:40:30)
t_{lag}	13.33±0.66 h	24.13±1.87 h	26.70±0.87 h	36.70±0.72 h
$t_{1/2}$	15.07±0.75 h	18.47±2.57 h	25.13±0.63 h	38.00±0.7 h

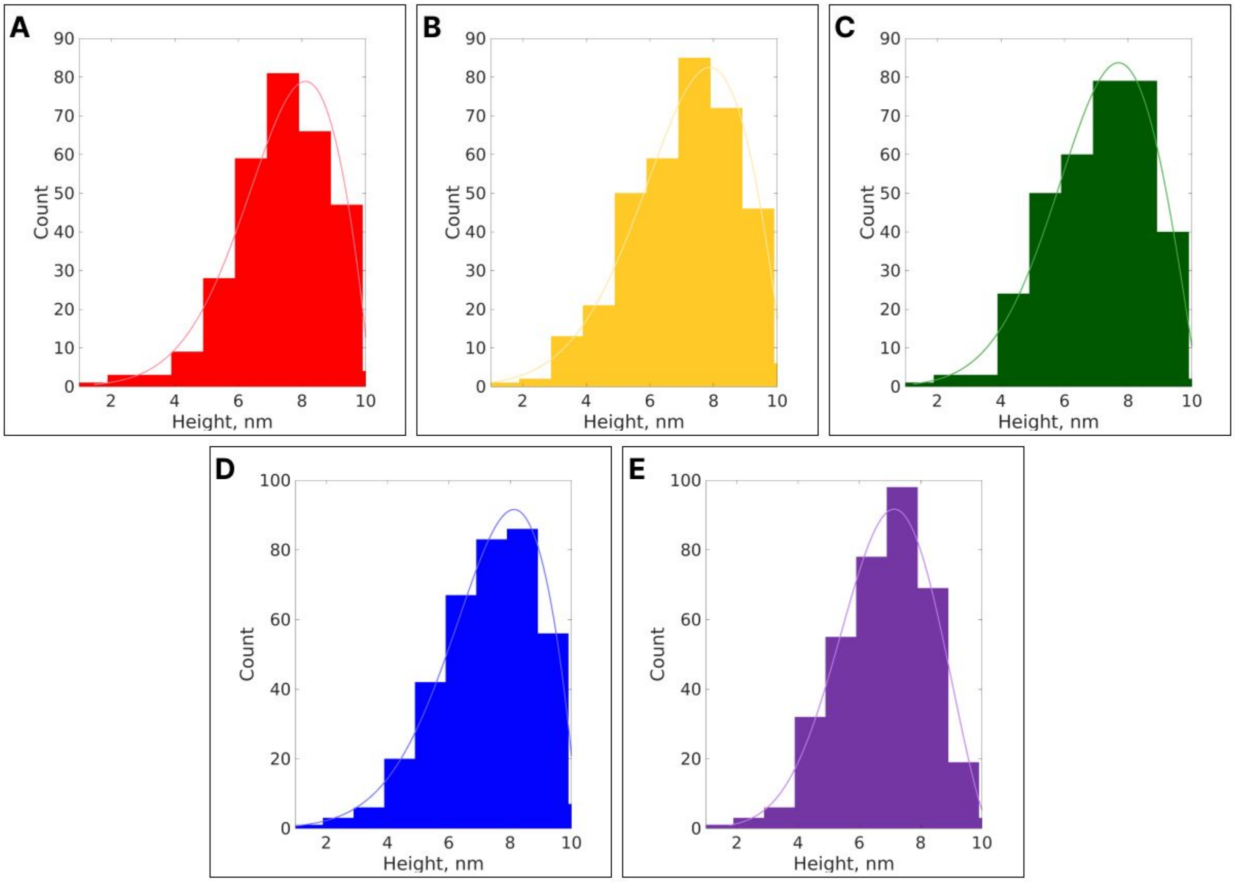

Figure S1. Height profiles of insulin aggregates formed in the lipid-free environment (A), as well as in the presence of PC:PE:PS (40:40:20) (B), PC:PE:PS (30:40:30) (C), PC:PE:PS (20:40:40) (D) and PC:PE:PS (30:30:40) (E).

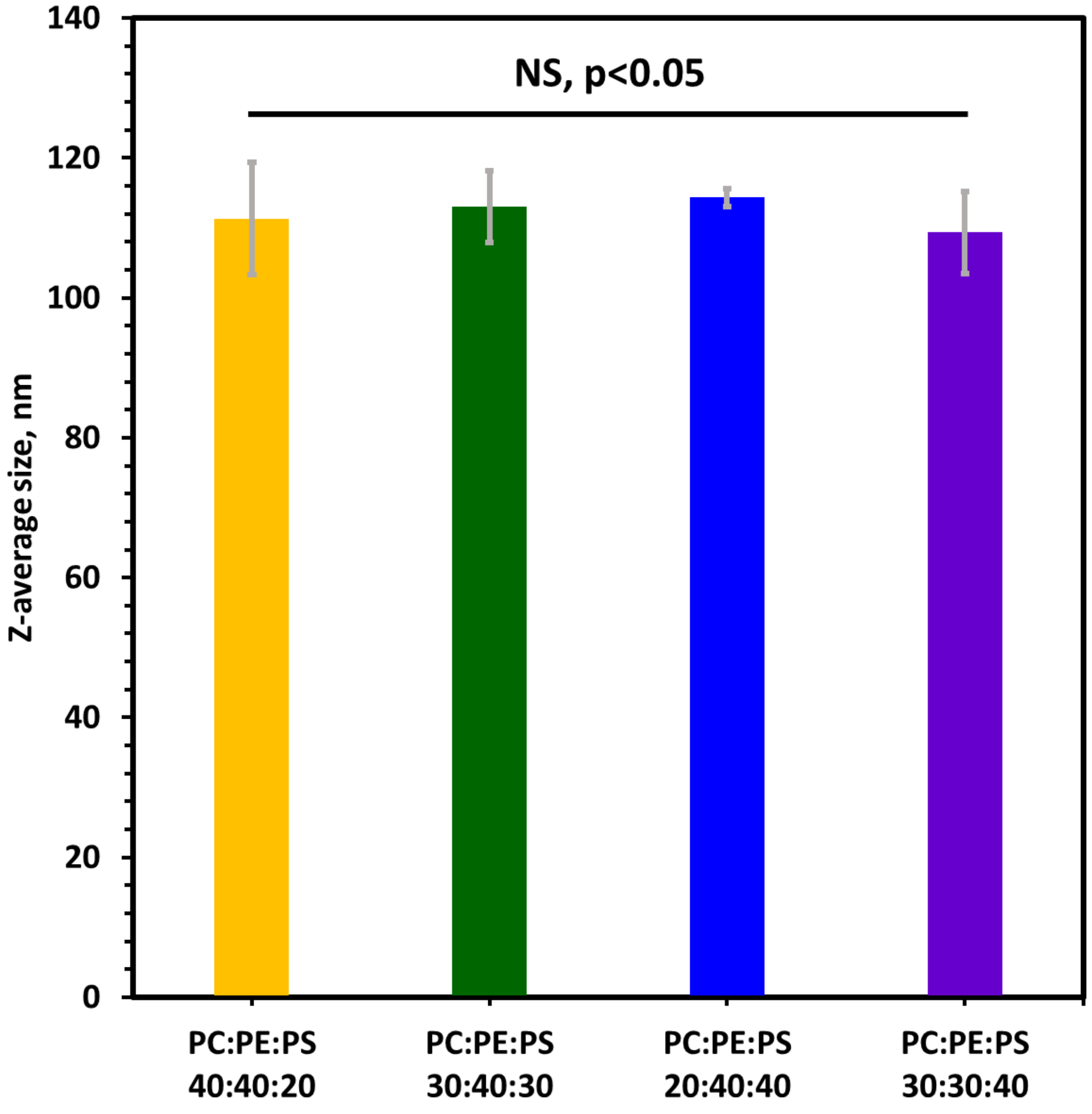

Figure S2. A histogram of diameters of LUVs determined using DLS.

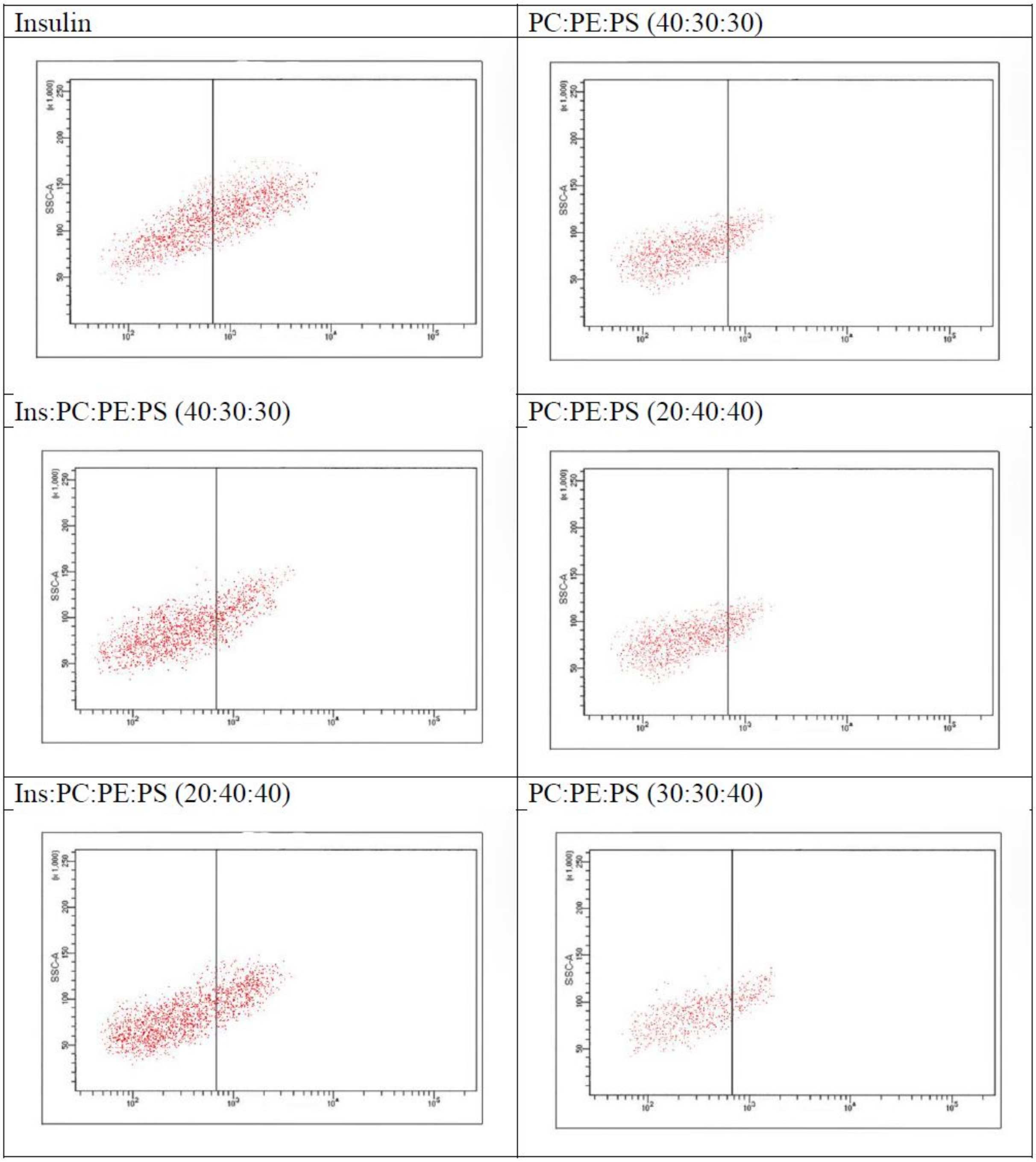

Figure S3. Flow cytometry plots of ROS measurements.

Ins:PC:PE:PS (30:30:40)

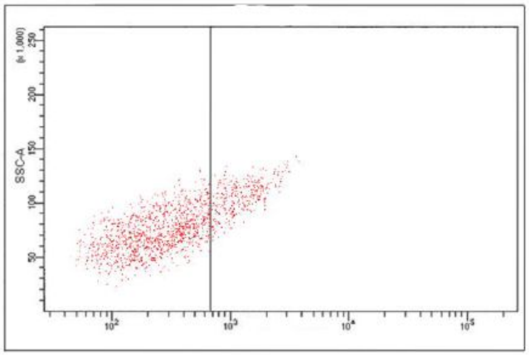

PC:PE:PS (40:40:20)

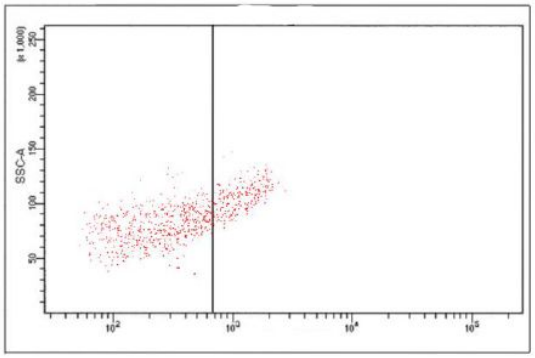

Ins:PC:PE:PS (40:40:20)

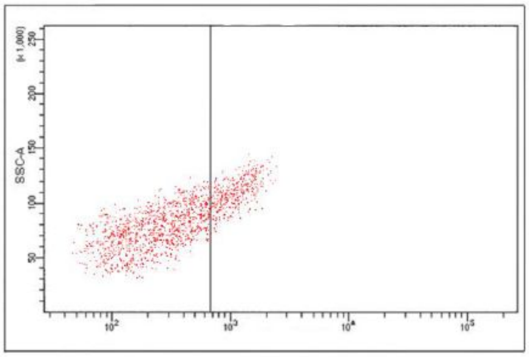

Control

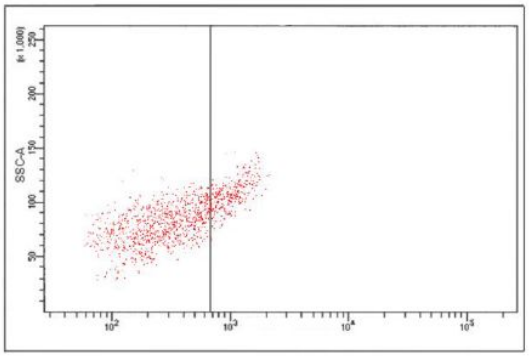

Figure S4. Flow cytometry plots of JC-1 measurements.
